# An Initial Psychometric Evaluation and Exploratory Cross-Sectional Study of the Body Checking Questionnaire among Brazilian Women

**DOI:** 10.1371/journal.pone.0074649

**Published:** 2013-09-11

**Authors:** Angela Nogueira Neves Betanho Campana, Viren Swami, Carolina Mie Kawagosi Onodera, Dirceu da Silva, Maria da Consolação Gomes Cunha Fernandes Tavares

**Affiliations:** 1 Department of Health Science, Sagrado Coração University, Bauru, Brazil; 2 Department of Psychology, University of Westminster, London, United Kingdom; 3 Department of Psychology, HELP University College, Kuala Lumpur, Malaysia; 4 Department of Physical Education, University of Campinas, Campinas, Brazil; 5 Department of Education, University of Campinas, Campinas, Brazil; Universidad Europea de Madrid, Spain

## Abstract

Body checking is considered an expression of an excessive preoccupation with appearance. The first aim of this study was to evaluate the psychometric properties of a Brazilian Portuguese version of the Body Checking Questionnaire (BCQ). Additionally, we wanted to examine the questionnaire’s associations with body avoidance behaviour, body mass index, dietary habits, and the intensity, frequency, and length of physical exercise. Finally, we also examined the differences between the total BCQ score and the individual BCQ factor scores. Differences between active and sedentary persons and between non-dieters and those on weight-loss diets were also analyzed. For the psychometric study, 546 female public university students from four different courses were surveyed. Two minor samples of university students and eating disorders women were also recruited. In the second part of the study, 403 women were recruited from weight-loss programs, gyms, and a university. All participants were verbally invited to participate in the research and voluntarily took part. Confirmatory factor analysis showed a good fit to the original model of the Brazilian BCQ that retained all 23 items. Satisfactory evidence of construct validity and internal consistency were also generated through analysis of factor loadings, *t*-values, Cronbach’s alpha, and construct reliability tests. The results also showed associations among body checking and body avoidance, body satisfaction, social anxiety, body mass index, and the frequency and intensity of physical exercise. Significant differences were found between non-dieters and weight-loss dieters for all BCQ factors and the total BCQ score. For physically active and sedentary persons, a significant difference was only observed for idiosyncratic checking behaviour. In conclusion, the BCQ appears to be a valid and reliable scale for Brazilian research, and the associations and differences found in this study suggest that women at gyms and especially in weight-loss programs should be targeted for future body checking studies.

## Introduction

Body-image disturbance is a multifaceted construct involving both perceptual and attitudinal components. These include feelings of having an inadequate body, anxiety about body shape or weight, preoccupation with the appearance of particular body parts, and cognitive distortions about body appearance or function [1]. Body checking and avoidance are behavioural manifestations of body image disturbance [2], [3]. These behaviours are adopted to accommodate a negative body image, a condition more distressing and inhibiting than body dissatisfaction [4]. Body checking and avoidance behaviours are recognized as the core manifestation of eating disorder psychopathology, also defined as the overvaluation of body shape and weight [5].

Body checking behaviour is characterized by an excessive preoccupation with appearance. This includes compulsive weighing, pinching of body parts, constant body comparisons with others, frequent checking of appearance in mirrors, and repeated ritualistic measurements. Body checking plays an ambiguous role in accommodating anxiety while simultaneously maintaining a negative body image [2], [6]. It is more prominent during periods of fasting and weight loss for eating disorder patients [6]. For female eating disorder patients, body checking and avoidance behaviors are highly correlated [7], [8], and both show significant correlations with overvaluing weight and shape [9]. In obese women and men, body checking may be a risk factor for eating disorders by magnifying perceived bodily imperfections which results in body dissatisfaction [10].

However, the role of body checking as a reinforcement of negative body evaluation does not occur only for eating disorders. In Western culture, the discrepancy between real and ideal body shape and type is a common experience for women [11]. Recently, there has been evidence that body checking behaviour could be also normative, particularly for young women [12]. One study has reported that body checking and avoidance behaviors are associated and that both are also associated with the fear of fat, body dissatisfaction, and lower self-esteem in control persons in weight loss programs [13].

In normal-weight men, body checking has been associated with increased shape and weight concerns, a desire to increase size and strength, negative beliefs about appearance, and functional impairments caused by bodily concern [14]. For women, the available research has shown that body checking behaviour is positively associated with increased upward social comparisons to media images [15], lower levels of feminist beliefs [16], body concern [6], obsessive-compulsive symptoms, perfectionism, negative [17] and narcissistic characteristics [18], body mass index, and age [19]. Known predictors of body checking behaviours are negative affect and obsessive-compulsive symptoms with the latter being relevant only for men [17]. Finally, body checking could also predicted the presence or absence of objective binging and purging in a non-clinical study [19].

The first measure developed to assess the body checking behaviour is the Body Checking Questionnaire (BCQ; [2]) which focuses on concerns and feelings related to body fat. The BCQ is especially used with female subjects since it does not measure muscularity, an important component of the male corporeal experience [20]. Typically, the BCQ has 23 items that are distributed in a structural model comprised of one second order factor (Body Checking) and three first order factors (Overall Appearance, Specific Body Parts, and Idiosyncratic Checking). Satisfactory evidence of internal reliability (α = .83–.90), convergent and discriminant validity, and indexes of adjustment (RMSEA = .076, CFI = .90, IFI = .90) were generated during the development of the BCQ [2]. BCQ has already been translated into and psychometrically validated for Italian [21], German [22], and Norwegian [23] languages. In Italy and Norway, comparable results were found for internal reliability (α between.84 to. 92 in Italy and.66 to 87 in Norway), and the structural model was also confirmed with equally satisfactory indexes of adjustment (Italy: RMSEA = .056, CFI = .97, IFI = .97; Norway: RMSEA = .056, CFI = .91, IFI = .90). In Germany, however, the second order factor of the structural model had no satisfactory adjustment (.082< RMSEA <.095; CFI = .88). An exploratory factor analysis was conducted, and a one factor solution explaining 48% of the variance was accepted [21]–[23].

The present study sought to examine body checking and its correlates among women in Brazil, the largest national economy in Latin America. Despite the fact that the Body Checking and Avoidance Scale (BCAQ) [24] and Body Checking Cognitions Scale (BBCS) [25] are already available in Brazilian Portuguese, they cover different aspects of the body checking construct [26] from those available in BCQ. BCCS covers the erroneous cognitions regarding accuracy and value of the body size, shape and weight, but not the presence or severity of body checking behaviours, as BCQ does [27]. BCAQ is a semi structured interview, specially developed for eating disorder patients. It assesses body checking and avoidance behaviors frequency and development, emotional response and the impact of them in eating control [6]. Therefore, we investigated the factor structure of the Brazilian Portuguese version of the BCQ and its construct validity. We also investigated the association of BCQ factor scores with previously established psychosocial and demographic variables such as age, body mass index, body avoidance behavior, and dietary habits. Physical exercise habits were also included in this analysis since Brazilian culture attaches extreme importance to a “well-shaped body” [28]. Young men and women are constantly aiming for the perfect body: fit, tan, and thin. How this is achieved, whether it is through diet, steroids, amphetamines, excessive exercise, or plastic surgery, is not important. A recent study found that 82% of young women (12 to 29 years of age) in south Brazil reported body dissatisfaction and that 65% of them wanted to lose weight [29].

Physical exercise is an important method of changing body shape with or without corresponding diet changes [30]. Studies have presented mixed findings on the association and relationship of physical exercise to body image variables. Some reports indicate a positive effect and association with a positive body image while others correlate physical exercise to body dissatisfaction, perfectionism, eating disorders, and social anxiety [31], [32]. Given these conflicting results, this current study of the association between physical exercise and body checking behavior could contribute to a better understanding of body image in Brazilian culture.

## Study 1: Confirmatory factor analyses, reliability, and validity

### Methods

This study was undertaken with the permission of Professor Williamson to translate and validate the BCQ into Brazilian Portuguese language. Ethical approval for this study was obtained from the ethics committee of the University of Campinas (number 0207.0.146.000-06). All participants provided written informed consent.

#### Participants

*Main non-clinical group*: A non-probabilistic sample of 546 women were recruited among students at a public university in the state of São Paulo. The students were from the departments of Education (34.9%), Physical Education (19.6%), Food Engineering (27.3%), and Nursing (18.2%). The ages of the participants ranged from 18 to 55 with 86.1% between 18 and 24. As determined by their body mass index (BMI), 80.3% had normal weight with only 11.9% above and 7.8% below the ideal weight. When asked about their health, 13.3% considered it excellent, 79.8% considered it good or very good, and only 6.8% found it regular or bad.

*Secondary non-clinical group*: A second sample of 14 university students answered a questionnaire. The mean age of these students was 22.38 years (*SD* = 5.82), and the mean self-reported BMI was 23.68 kg/m^2^ (*SD* = 4.56). This second sample was used to evaluate discriminant validity.

*Clinical group*: Fourteen patients with eating disorders were recruited from the Eating Disorders Outpatients Clinic for the discriminant validity analysis. The mean age of these patients was 20.92 years (*SD* = 5.82), and the mean self-reported BMI was 20.26 kg/m^2^ (*SD* = 2.64).

All participants participated on a voluntary basis without any kind of remuneration.

#### Study design, materials and procedures

In order to produce an idiomatic, semantic, culturally, and conceptually equivalent version of the BCQ in Brazilian Portuguese, five distinct steps were used [33]. First, the BCQ was independently translated into two Brazilian Portuguese versions (T_1_ and T_2_) by two native Portuguese speakers. Second, a synthesized translation (T_12_) was drawn up by the two translators and a neutral judge. Third, from the synthesized versions, two back-translations (BT_1_ and BT_2_) were created by two translators (English-speaking natives with Brazilian Portuguese proficiency) who had no knowledge of the original BCQ or body image aspects. Fourth, all the versions (T_1_, T_2_, T_12_, BT_1_, BT_2_) were forwarded to an Expert Committee consisting of the two translators, the two back-translators, the judge of the synthesized version, a psychoanalyst (with clinical experience in eating disorder treatment), a methodologist, and a linguist. This committee examined each version of the questionnaire and discussed the items to ensure a clear pretest version that was equivalent to the original in terms of semantics, language, culture, and concepts. Finally, we conducted a pretest.

The BCQ version approved by the Expert Committee was given to 27 female Physical Education undergraduate students, aged 18 to 21. They approved the new layout, preferred the specific action verbs, and expressed doubts on item 18. Due to this doubt, one of the BCQ author’s, Professor Williamson, was contacted, and according to his directions, the confusing item was modified, and a new pretest was conducted. For the second pretest, a group of 7 female post-graduate students, aged 20 to 35, from the Adapted Physical Education department was interviewed for the purpose of verifying the adequacy of the new version. Based on their feedback, we were certain that this version of the questionnaire was clear and understandable.

The following scales were used in this study:

*Body Checking Questionnaire* (BCQ; [2]): The 23-item BCQ was developed as a measure of the association between body checking behaviour and body fat. Items were rated on a 5-point Likert-type scale (1 = *Never*, 5 = *Very Often*). No items were reverse-coded prior to the analyses. Reas et al. (2002) proposed a higher-order construct comprised of a three second-order factors: Overall Appearance Checking (OAC; items 3, 5, 8, 11, 12, 13, 15, 17, 21, and 22), Specific Body Parts Checking (SBP; items 1, 2, 6, 9, 10, 14, 16, and 19), and Idiosyncratic Checking (IC; items 4, 7, 18, 20 and 23). Posterior analysis confirmed this as the original factor structure for the BCQ [9], [21], [23].

*Brazilian Body Image Avoidance Questionnaire* (BIAQ; [3]; Brazilian version; [34]): The BIAQ is a measure of body avoidance behaviour, especially those related to descriptions of food and the body. The best fit for the Brazilian Portuguese version of BIAQ (RMSEA = .068, NFI = .97, NNFI = .97, CFI = .98, GFI = .94, AGFI = .91, *^2^/df = *3.43) was achieved with a 13-item model and three factors: hunger control strategies and body shapes (CS), refusal strategies (RS), and body exposure and accommodation strategies (AS) [34]. Reliability was.85 for the primary non-clinical group,.83 for the secondary non-clinical group, and.86 for the clinical (eating disorder) group.

*Demographics:* Participants self-reported their demographics such as age, height, and weight. The latter two variables were used to calculate BMI.

To collect data, the researchers visited each room of four undergraduate courses: Physical Education, Nursing, Education, and Food Engineering. The questionnaires were answered in the classroom, during the class interval, and in the presence of the researchers. At the Eating Disorders Outpatients Clinic, the head of Psychiatry presented the researchers to the patients, and the study objective was explained verbally to all of them. The questionnaires where then answered at separated desks in a common room. A consent form explaining the procedures and objectives of the study was read and signed by all participants. Each participant took approximately 15 minutes to complete the questionnaire. Once again, participants participated on a voluntary basis without any kind of remuneration.

#### Statistical analysis

The multivariate method of confirmatory factor analysis (CFA) was used to determine the validity and reliability of the questionnaire. The *listwise* deletion criterion was adopted for missing data, and due to the lack of multivariate normality in our data, the Unweighted Least Square method of extraction was used [35]. The following indexes were considered for model adjustments: Goodness-of-Fit Index (GFI), Adjusted Goodness-of-Fit Index (AGFI), Normed- Fit Index (NFI), Non-normed-Fit index (NNFI), and Comparative Fit Index (CFI). According to the literature, it is recommended that these indexes be equal to or above.90 [36]. The Root Mean Square Error of Approximation (RMSEA), with an established acceptance value of below.08, was also considered [37].

For internal reliability, Cronbach’s alpha and construct reliability [36] were evaluated. Convergent validity was verified from the *t*-values and factor loadings of observable variables. Acceptable indicators were those *t*-values ≥1.96 [38], and because the study included more than 350 participants, factor loadings >.30 were also acceptable [36]. As an additional analysis of convergent validity, the association with body avoidance score was also tested. Discriminant validity was analyzed using the score differences between the eating disorder and secondary non-clinical groups.

The unidimensionality of each factor was also analyzed in order to evaluate the quality of the observed variables (items) and to explain the latent variable (factor). Residuals were included among the observed variables for the model. Residuals must be less than ±2.58 in order to state that the items represent only one factor.

CFA was conducted with the LISREL® 8.51 software. SPSS 15.0 was used for correlational and variance analysis.

### Results

A Kruskal-Wallis test was conducted to evaluate differences between the participants of each undergraduate course (Physical Education, Nursing, Education, and Food Engineering). The results of the analysis indicated that there was not a significant difference in the median OAC [*^2^*(3, *N = *546) = 4.16, *p = .*24], SBP [*^2^*(3, *N = *546) = 6.96, *p = .*07], nor IC [*^2^*(3, *N = *546) = 5.42, *p = .*14]. Consequently, we treated these samples as a single group in all subsequent analyses.

#### BCQ factor structure

**Figure 1:**
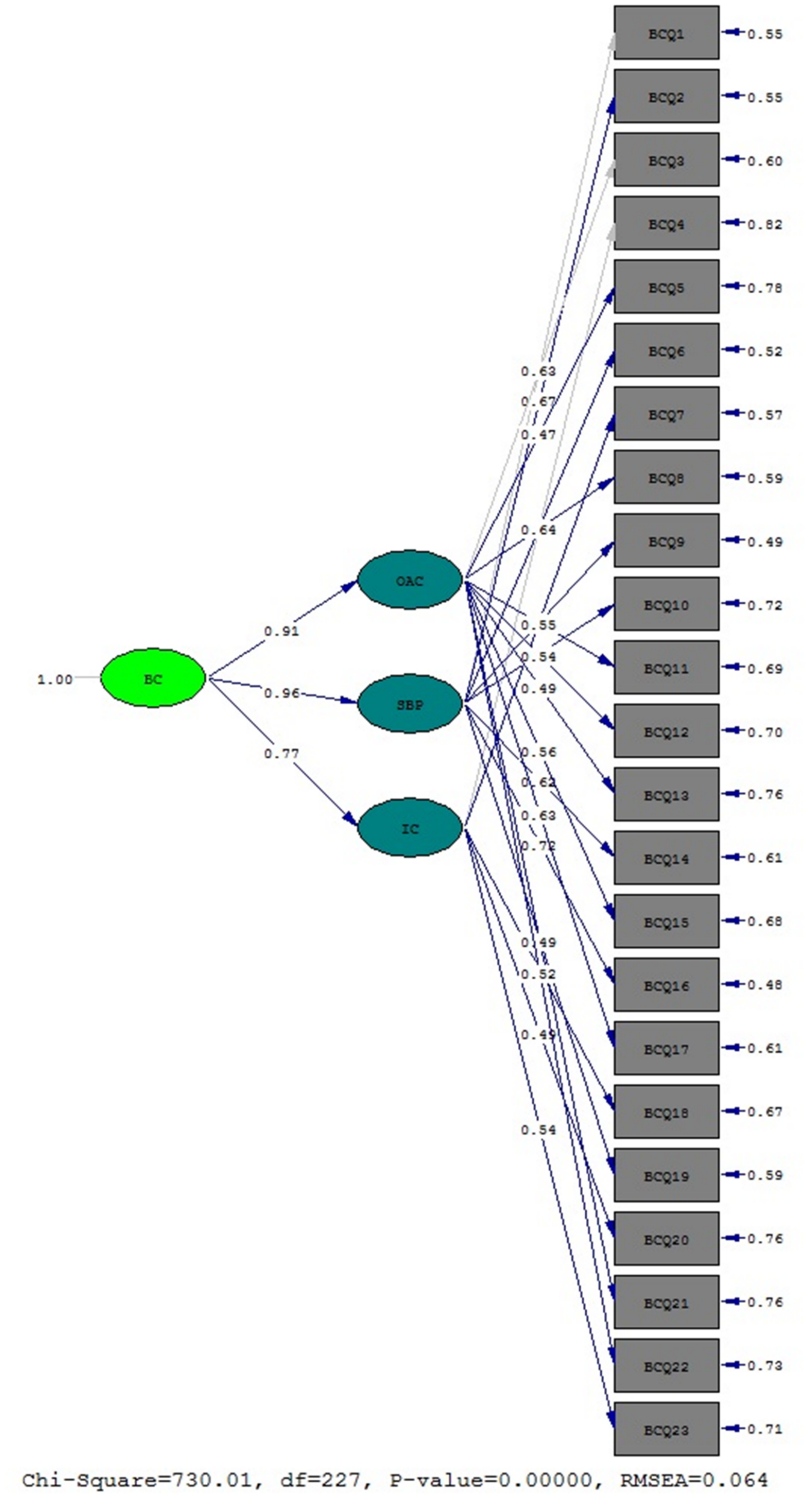
Adjusted model for Brazilian version of Body Checking Questionnaire.

The original BCQ model was comprised of one second order factor (Body Checking) and three first order factors (OAC, SBP, and IC). The original model was a good fit for the Brazilian data (*^2^* = 730.01, *p*<.001; RMSEA = .064, GFI = .98, AGFI = .98, NFI = .97, CFI = .99, NNFI = .99, *^2^/df = *3.21) (Figure 1). These parameters were similar to previous psychometric studies, including the BCQ development studies and those of other Western countries (Table 1). No items were excluded, and given the size of the sample, all factorial loadings were adequate, even when compared to other Western cultures (Table 2). Additionally, the structural coefficients of the second order and first order latent variables (OAC, SBP, and IC) were all high and statistically significant (*r = *.91,.96,.77, respectively; *p*<.001).

**Table 1:** BCQ fit indexes from actual and previous psychometric evaluations.

Country	χ^2^/df	RMSEA	IFI	NFI	NNFI	CFI	GFI	AGFI	M	SD
**Brazil**	3.21	.064	–	.97	.99	.99	.98	.98	48.87	14.88
**USA** 1	–	.076	.90	–		.90	–	–	56	16
**Italy** 2	2.75	.056	.97		.97	.97	–	–	44.2	14.7
**Germany** 3	3.32	<.082				.88				
**Norway** 4		.056	–	.90		.91	–	–	45.6	13.3

Note. χ^2^/df = Chi – Weighted Square, RMSEA = Root Mean Square Error of Approximation; IFI = Incremental Fit index; NFI = Normed Fit index; NNFI = Non-normed Fit index; CFI = Comparative fit index; GFI- Goodness-of-fit; AGFI = Adjusted Goodness-of-fit; *M = *mean; *SD* = standard deviation.

1 [2].

2 [21].

3 [22].

4 [23].

**Table 2:** Factor loadings e *T-values* from the present and factor loadings from previous psychometric evaluations.

		USA^1^	Italy^2^	Brazil	
Factors	Items	Factor Loading			*T-value* (*p*)
					–
	**3**	.69	.76	.63	–*
	**5**	.62	.70	.47	15.23 (.03)
	**8**	.76	.82	.64	17.09 (.03)
	**11**	.63	.81	.55	16.71 (.03)
**OAC**	**12**	.64	.63	.55	15.87 (.03)
	**13**	.67	.78	.50	15.56 (.03)
	**15**	.58	.78	.57	16.05 (.03)
	**17**	.69	.72	.63	17.49 (.03)
	**21**	.69	.74	.49	15.35 (.03)
	**22**	.63	.61	.52	16.08 (.03)
	**1**	.79	.83	.67	–*
	**2**	.69	.77	.67	19.53 (.03)
	**6**	.70	.81	.69	18.77(.03)
**SBP**	**9**	.83	.86	.71	19.49(.03)
	**10**	.75	.82	.53	17.28 (.03)
	**14**	.85	.84	.62	18.92 (.03)
	**16**	.77	.86	.72	19.79 (.03)
	**19**	.73	.67	.64	18.70 (.03)
	**4**	.85	.84	.43	–*
	**7**	.61	.76	.66	7.52 (.06)
**IC**	**18**	70	.74	.57	7.77 (.07)
	**20**	.80	.87	.49	6.84 (.03)
	**23**	.68	.81	.54	7.78 (.06)

**Note:** OAC** = **Overall Appearance Checking factor; SBP = Specific body parts factor; IC = idiosyncratic factor. Factor loading were not reported in the German [22] and Norwegian study [23].

* fixed parameters.

1 [2].

2 [21].

#### Unidimensionality

Only.01% of residuals were above the reference value of ±2.58 (CFI = .99). The highest positive residual was 11.54 and occurred between items 6 and 7. The highest negative residual was −3.62 and occurred between items 22 and 7. Nevertheless, the factor loadings of items 6, 7, and 22 were high (.69,.66, and.52, respectively), indicating their relevant contribution to their respective factors.

#### Internal consistency, convergent and discriminant validity

Cronbach’s alpha coefficients indicated high levels of internal consistency for OAC (α = .83) and SBP (α = .87) and an acceptable value for IC (α = .70). The construct reliability coefficients indicate an unsatisfactory level of internal reliability for IC (CR = .67). The test also confirmed the high levels of internal reliability for OAC (CR = .86) and SBP (CR = .82) seen by Cronbach’s alpha.

Convergent validity was analyzed through factor loading, *t*-values, and correlations between the total BCQ score and the BIAQ factor scores, age, and BMI. All factor loadings were above.04, and all *t-*values were above 1.96 and statistically significant (Table 2). The Spearman test indicated a weak but significant correlation between BCQ score and the AS of the BIAQ (*r_s_* = .08, *p* = .05) and between the BCQ score and age (*r_s_* = −.12, *p*<.001). No other correlations were significant.

To determine discriminant validity, we checked the score difference between the eating disorder (*N* = 14) and secondary (non-clinical) control (*N* = 14) groups. The *t-*test indicated that participants with an eating disorder (*M = *77.75, *SD* = 20.33) had significant higher scores on the BCQ than the controls (*M = *48.28, *SD* = 13.68) [*t*(26) = 4.47, *p<*.001, *d* = 1.69].

### Discussion

The original BCQ model achieved good global goodness-of-fit indexes, indicating a satisfactory application in Brazil. These indexes are similar to the adjustment indexes of the original questionnaire and its validations [2], [6] and were specifically close to the adjustment values of the Italian [21] and Norwegian versions [23]. Additionally, the BCQ first order factor scores showed satisfactory evidence of convergent validity. Regarding discriminant validity, despite the fact that a significant difference between the criteria groups was established, findings should be consider carefully, because of the small size of the clinical group. The unidimensionality of the factors were also successfully verified. Despite its higher negative and positive residuals, item 7 did not compromise the questionnaire. We decided not to eliminate this item in order to keep the BCQ’s original factor structure which is an advantage for transcultural studies.

The factor loadings for the Brazilian BCQ were all above the acceptable value for this sample size, and 19 items had a factor loading above 0.50. However, the factor loadings for the Brazilian BCQ were generally smaller than those of the development study [2] and the Italian version [21]. Because we took careful steps to ensure that the BCQ was reliably translated, it is unlikely that a translational issue caused this problem. It is more plausible that cultural differences led to this situation. Specifically, it is possible that some aspects of the female corporeal experience, specific to Brazil, were not considered by the BCQ. This may, consequently, impact body checking behaviour.

IC did not attain the minimal value of internal reliability in the construct reliability test (CR ≥.70). However, because adequate values were found with the Cronbachs alpha test for all first order factors, we maintained this factor in the total BCQ score. Additionally, the results replicated those from previous works and confirmed the theory that Body Checking is a higher order construct comprised of three interrelated constructs.

It is also worth considering the correlations found between age and the BIAQ AS and the BCQ score. Although these correlations were weak, it should be noted that this is consistent with previous studies (e.g. [2], [7], [9], [10]). Unlike these same studies, however, no associations were found between the BCQ and BMI. Because the concept of a fit body in Brazil is broader than just an adequate weight for height [28], we speculate that, in this particular group, other body image traits could be associated with the BCQ, including body dissatisfaction and the desire to be thin. Not considering these aspects in the questionnaires was a clear limitation of this study.

Other limitations in this study must be considered. First, despite our efforts to collect data from different sets and avoid the majority of university participants, our sample is not representative of the general population in Brazil. Therefore, all results found here should not be generalized. Second, because no eating disorder screening instrument was used, potentially undiagnosed eating disorders, either clinical or subclinical could be present in the main non-clinical and at the second non-clinical sample. Given, the size of the main sample and the significant difference showed between the clinical and the non-clinical sample, is unlikely that the results were affected, however, a future research should confirm this. Third, we have only validated BCQ scales for a specific group of Brazilian women, and the clinical sample used for discriminant validity was quite small. Future studies should also examine the psychometric properties of the Brazilian BCQ among specific samples in which checking behaviour has a prominent importance, both for its function (e.g. burn patients in rehabilitation) and its appearance (e.g. gymnasts, athletes in general).

## Study 2: Associations and differences among body checking behaviour in Brazilian women

### Methods

Ethical approval for the second part of this study was also obtained from the ethics committee of the University of Campinas (number 0198.0.146.000–11). Again, all participants provided written informed consent.

### Participants

A total of 403 participants, recruited in different settings such as at weight watching programs, gyms, and university, answered the questionnaire. The ages of the participants ranged from 18 to 71 years (*Mdn = *26), and the median self-reported BMI was 22.75 kg/m^2^ (range = 23,14). Only 8.9% of the participants were considered obese, and 27.7% were overweight. The majority (64.8%) of the participants described themselves as physically active. When questioned about their eating habits, 3.2% were on diets to gain weight, 41.8% were on diets to lose weight, and 55.1% were not on any kind of diet.

#### Study design and procedures

The researchers recruited participants directly through opportunistic sampling at weight watching meetings, gyms, and a physical education school. The objectives of the study were verbally explained, and a consent form explaining the procedures and objectives of the research study was read and signed by all volunteers. All participants completed anonymous paper-and-pencil versions of the questionnaire at a quiet location before returning their questionnaires to the researcher. Participation was on a voluntary basis, and participants did not receive any form of remuneration. Each volunteer took approximately 20 minutes to complete the questionnaire.

#### Materials

*Brazilian version of the Body Checking Questionnaire* (BCQ; [2]): The 23-item Brazilian BCQ, as described in Study 1, was used. For this sample, Cronbach’s alpha coefficients indicated high values of internal consistency for OAC and SBP of.82 and.84, respectively. The internal reliability value of IC was lower with a value of.70. The model found in Study 1 was replicated in this sample with satisfactory fit indexes (*^2^* = 726.66, *p*<.001; RMSEA = .074, GFI = .97, AGFI = .97, NFI = .96, CFI = .98, NNFI = .98, *^2^/df = *3.2).

*Brazilian version of the Body Image Avoidance Questionnaire* (BIAQ; [3]; Brazilian version [34]): The 13-item version of Brazilian BIAQ, as described in Study 1, was used. The BIAQ had acceptable reliability for all factors with.75 for CS,.73 for RS, and.71 for AS. The previous Brazilian BIAQ model was also replicated in this sample with a good adjustment (*^2^* = 292.56, *p*<.001; RMSEA = .072, GFI = .93, AGFI = .90, NFI = .97, CFI = .97, NNFI = .97, *^2^/df = *3.92).

*Single–item measures:* Two single-item measures, as follows, about body satisfaction and social anxiety were also included: “On a 1 to 10 scale, in which 1 = ‘not at all satisfied’ and 10 = ‘very satisfied’, how do you classify your current satisfaction with your body?” and “On a 1 to 10 scale, in which 1 = ‘very anxious’ and 10 = ‘not at all anxious’, how do you classify your feeling of anxiety when you need to present yourself to the public (e.g. for a speech)?”. Because of the unambiguous nature of the construct evaluated by these two questions, these single-item measures were considered acceptable [39]. We also chose this approach to promote the participants willingness to answer the questionnaires, especially at gyms and weight watching meetings since a smaller questionnaire could be answer without taking much of their time.

*Demographics:* Participants self-reported their demographics, namely age, weight, height, and dietary habits. To evaluate their physical exercise habits, participants completed the Kasaris Fit Index Scale [40] in which they self-reported their physical exercise practice in terms of frequency (1 = ‘<once a month’, 5 = ‘>6 times per week’), intensity (1 = ‘Light aerobic exercise’, 5 = ‘High intensity activities’), and duration (1 = ‘<10 minutes per session’, 5 = ‘>30 minutes per session’).

### Results

#### Initial results and correlations

The Kolmogorov-Smirnov test was use to evaluate data distribution. Because of the non-adherence to normal distribution of the data, specifically for the body checking and avoidance factor scores, non-parametric tests (Spearmann correlation and the Mann-Whitney U test), were used to analyse the data. Descriptive statistics (Median, and Range) of all participants for all variables included in this study and their correlations are reported in Table 3. Higher scores in the BIAQ factors (*r_s_* = .48–.38), physical exercise intensity (*r_s_* = .16), social anxiety (*r_s_* = .13), and lower body satisfaction (*r_s_* = −.32) were significantly correlated with higher OAC scores from the BCQ. We also found positive correlations of the BCQ SBP with the BIAQ factors (*r_s_* = .42–.31), social anxiety (*r_s_* = .13), frequency (*r_s_* = .11), and intensity (*r_s_* = .15) of exercise practice. The BCQ SBP was also negatively correlated with body satisfaction rank (*r_s_* = −.32). The BCQ IC had a positive association with only the BIAQ factors (*r_s_* = .42–.31) and a negative association with body satisfaction rank (*r_s_* = −.26) (Table 3).

**Table 3:** Descriptive statistics for participants and Spearman correlations among physical exercise habits and scales responses.

Item	*Mdn*	*R*	(1)	(2)	(3)	(4)	(5)	(6)	(7)	(8)	(9)	(10)	(11)	(12)	(13)	(14)
(1) BCQ total score	199	256	–	.92**	.91**	.74**	.55**	.42**	.34**	.48**	−.32**	.13**	.11	.16**	−.04	.26**
(2) BCQ-AOC	47	52		–	.73**	.59**	.54**	.43**	.30**	.48**	−.26**	.12*	.11	.18**	.002	.19**
(3) BCQ-SBP	40	51			–	.57**	.49**	.38**	.31**	.42**	−.32**	.13**	.12*	.15*	−.06	.26**
(4) BCQ-IC	14	22				–	.37**	.24**	.29**	.32**	−.26**	.05	.02	.02	−.05	.29**
(5) BIAQ total score	74	69					–	.78**	.62**	.83**	−.47	.14**	.03	.07	−.03	.49**
(6) BIAQ-RS	13	20						–	.16**	.57**	−.27**	.06	.07	.14**	−.01	.30**
(7) BIAQ-CS	36	34							–	.41**	−.43**	.19**	−.07	−.09	−.01	.34**
(8) BIAQ-AS	25	28								–	−.40**	.10*	.04	,07	−.04	.43**
(9) Body satisfaction	7	9									–	.22**	.03	.12*	.04	−.54**
(10) Social Anxiety	5	9										–	−.01	.06	.05	−.07
(11) PE Frequency	4	3												.46**	.22**	.01
(12) PE Intensity	2	4												–	.17**	−.05
(13) PE Length	4	4													–	−.08
(14) BMI	22,74	43,14														–

**Note:** *Mdn* = median, *R* = range, BCQ = Body checking Questionnaire, OAC** = **Overall Appearance Checking factor; SBP = Specific body parts factor; IC = idiosyncratic factor, BIAQ = Body Image avoidance Questionnaire, RS = refusal strategies factor, CS = control strategies factor, AS = accommodation strategies factor, PE = Physical Exercise, BMI = body mass index.

#### Group differences: dieter *vs.* non-dieters

Previous works consistently reported differences among female dieters and non-dieters [2], [7], [23]. Our results suggest that the same pattern exists among Brazilian women. Significant differences were found for total BCQ and BIAQ scores and in every factor of both questionnaires. The dieter group had higher OAC, SBP, and IC scores for the BCQ and higher RS and AS scores for the BIAQ (Table 4).

**Table 4:** Group differences: dieters *vs*. non-dieters.

		Dieters	Non-dieters				
Variable		*(n = *168)	(*n* = 222)	*U*	*Z*	*p*	*r*
BCQ-AOC	*Mean rank*	232.69	167.36	12400.5	−5.67	<.001	.29
BCQ-SBP	*Mean rank*	236.34	164.6	11787.5	−6.23	<.001	.32
BCQ-IC	*Mean rank*	231.24	164.60	12644.5	−5.56	<.001	.28
BCQ total score	*Mean rank*	239.18	162.44	11309	−6.66	<.001	.34
BIAQ-RS	*Mean rank*	233.14	167.02	12324.5	−6.09	<.001	.31
BIAQ-CS	*Mean rank*	247.68	156.01	9882	−7.97	<.001	.40
BIAQ-AS	*Mean rank*	258.29	147.98	8099	−9.61	<.001	.49
BIAQ total score	*Mean rank*	265.86	142.25	68227	−10.73	<.001	.54
Body satisfaction	*Mean rank*	139.28	232.05	9203	−8.07	<.001	.41
Social anxiety	*Mean rank*	186.43	202.36	17125	−1.39	.16	.07

Note: BCQ = Body checking Questionnaire, OAC** = **Overall Appearance Checking factor; SBP = Specific body parts factor; IC = idiosyncratic factor, BIAQ = Body Image avoidance Questionnaire, RS = refusal strategies factor, CS = control strategies factor, AS = accommodation strategies factor, Body satisfaction = In a 1 to 10 scale, in which 1 = not at all satisfied and 10 = very satisfied, how do you classify your currently body satisfaction?, Social anxiety = In a 1 to 10 scale, in which 1 = very anxious and 10 = not at all anxious, how do you classify your feeling of anxiety when you need expose yourself in public, for a speech, for example?

#### Group differences: physically active *vs.* sedentary

Because physical activity is also an important method of changing body weight and shape, we investigated differences in body avoidance and checking behavior among those who were physically active and those who were sedentary. A significant difference was observed for idiosyncratic checking behaviour (*U* = 14108, *p* = .01, *r = .*12). The sedentary group (*Mdn* = 223,94) had higher IC scores than the physically active group (*Mdn* = 192,18) (Table 5).

**Table 5:** Group differences: sedentary *vs.* physically active persons.

		Sedentary	Physically active				
Variable		*(n = *117)	(*n* = 286)	*U*	*Z*	*p*	*r*
BCQ-AOC	*Mean rank*	198.89	202.59	16447.5	−.29	.77	.01
BCQ-SBP	*Mean rank*	209.6	198.14	15800.5	−.90	.36	.04
BCQ-IC	*Mean rank*	223.94	192.18	14108.5	−2.55	.01	.13
BCQ total score	*Mean rank*	207.16	199.15	16088	−.63	.52	.03
BIAQ-RS	*Mean rank*	205.37	199.89	16229	−.46	.65	.02
BIAQ-CS	*Mean rank*	202.52	201.08	16636	−.11	.91	.01
BIAQ-AS	*Mean rank*	210.87	197.61	15650	−1.05	.29	.05
BIAQ total score	*Mean rank*	210.03	197.96	15750	−.95	.34	.05
Body satisfaction	*Mean rank*	162.02	218.35	12053.5	−4.46	<.001	.22
Social anxiety	*Mean rank*	177.71	211.94	13889	−2.69	.01	.13

Note: BCQ = Body checking Questionnaire, OAC** = **Overall Appearance Checking factor; SBP = Specific body parts factor; IC = idiosyncratic factor, BIAQ = Body Image avoidance Questionnaire, RS = refusal strategies factor, CS = control strategies factor, AS = accommodation strategies factor, Body satisfaction = In a 1 to 10 scale, in which 1 = not at all satisfied and 10 = very satisfied, how do you classify your currently body satisfaction?, Social anxiety = In a 1 to 10 scale, in which 1 = very anxious and 10 = not at all anxious, how do you classify your feeling of anxiety when you need expose yourself in public, for a speech, for example?

### Discussion

The results of the present study showed the expected associations of the total BCQ score and factor scores, the total BIAQ and factor scores, and BMI already seen in previous studies [7], [13], [41]. The association between the general BCQ score and the OAC and SBP scores with exercise intensity was new, however, and in the case of the latter, an association with exercise frequency was also found. Otherwise, no significant correlations were found for any BIAQ factors. These results suggest the existence of a feed-back system where the efforts for achieving the perfect lean body could magnify body surveillance as well as increased commitment to a physical exercise routine.

On the other hand, a significant difference was observed for only idiosyncratic body checking between sedentary and active persons. This difference suggests that those who exercise regularly have lower levels of this type of body checking. In fact, lower levels of anxiety, depression, and negative effect can be found in physically active persons [42]. Additionally, it has already been proposed that physical exercise increases positive body image, probably due to the sense that the body is improving through the physical exercise [43]. Consequently, the disposition to idiosyncratically check his or her body to assure that the body is under control could be lower in active persons because, in a way, there is already background assurance that the body is under control. However, we must not ignore the facts that no significant differences were found for overall appearance checking and specific body parts checking. This suggests that this sense of improving the body could not be a factor for reducing all aspects of checking behaviour. Given the contradictory findings of a previous study regarding physical exercise and body image traits [31] and these findings on the difference between sedentary and active persons, we should be looking at body checking behaviour more closely during exercise.

As expected, significant differences for total BCQ and BIAQ and their factor scores were found between dietary habits. It is worth mentioning the prevalence of normal weight participants (54.7%) and non-dieters (55.1%) in the sample, however. This evidence suggests that, for Brazilian women, weight loss dieting is accompanied by the highest levels of body checking and avoidance behaviours. Dieting is the first order choice in losing weight to achieve a beautiful body among Brazilians and commonly occurs without professional help [44]. However, despite the importance of dieting in Brazil, these results do not seem to be a cultural characteristic. In Western cultures, several studies also found higher levels of body avoidance and body checking behaviour in individuals who were dieting [2], [7], [23], [45].

Studies in other regions have also shown that dieters tend to be more distractible, anxious, depressed, unhappy, and preoccupied with food than their non-dieting counterparts [46]. Therefore, evidence of these characteristics should also be examined since those on diets give up the essential pleasure of eating freely, a pleasure connected with the basic needs for living [47]. The higher levels of body checking could be viewed as expected behaviour then since the expectation of achieving a beautiful, thin body and, therefore, eating normally again may stimulate a person to collect information on the progress of the effort. On the other hand, body avoidance among dieters could be viewed as an adaptive behaviour that protects the person when no weight loss is achieved in order for him or her to stay motivated and continue food restraint. Of course, this interpretation should be more examined more closely in a longitudinal and preferably multi-center study.

A number of limitations in this second study must also be considered. As the case of the first study presented early, our sample is not representative of the general population in Brazil, and hence, all results found here should not be generalized. Second, this study has a correlational design which precludes causal conclusions. Additionally, because of the non-normal distribution of the data, regression analysis could not be conducted, limiting our findings regarding predictors of body checking an avoidance behaviour to just our sample. Third, we did not concurrently measure the participants’ levels of drive for thinness and internalization of the thin ideal which has already been established as a mediator between sociocultural pressures of the ideal body and body dissatisfaction [48]. Given that previous evidences has shown that the latter is a predictor of body checking behaviour [17] and that body dissatisfaction is a widespread experience in Western culture [48], future research should account for this variable. Finally, the study design was also limited since single item measures were used to evaluate body satisfaction and social anxiety. Although this fact is not a fatal error for narrow constructs [38], an attitudinal scale could more deeply explore the associations between social anxiety, body satisfaction, and body checking behaviour.

## General Conclusions

This study showed satisfactory initial psychometric evidence of internal reliability and construct validity for the Body Checking Questionnaire in a sample of Brazilian women. We also investigated correlations between BMI, a direct measure of body satisfaction, social anxiety, and physical exercise habits with body avoidance and body checking behaviour. The results suggest a positive association between BMI and social anxiety with BCQ and BIAQ factors as well as a negative association of these variables with body satisfaction. Significant differences in BCQ and BIAQ factors were also observed between those dieting to lose weight and non-dieters. These finding replicated previous studies conducted in Western cultures.

In an innovative approach, this study analyzed physical exercise habits in the context of body image behavior in Brazilian women with mixed results. The results indicated a positive association between exercise intensity with overall appearance and specific body parts checking behaviour with the latter also associated with exercise frequency. Physically active persons had significantly lower scores for idiosyncratic body checking behavior, however. Hence, while two variables body checking behaviour could increase as physical exercise intensity and frequency increase, a peculiar factor of body checking may actually be lower in physically active people. This group also showed significantly higher scores for body satisfaction and lower scores for social anxiety. The nature of the influence of physical exercise on body image is complex and sometimes controversial [43]. Therefore, questions about the role of physical exercise in body image behavior remain.

The limitations of both studies were already presented in their respectively discussion. However, we must highlight here that future research should investigate the role of body checking and body avoidance behaviour as predictors of more general constructs more deeply in different Western and non-Western cultures. These constructs include, for instance, quality of life [41] and attentional bias [49]. On the other hand, these investigations should also research additional predictors of body checking and body avoidance as well as extend the actual knowledge of important triggers for body image dissatisfaction related behaviours. These triggers include compulsive symptoms [17], the comparison process [15], body-related self-critical thinking, and fear of fatness [12].

Despite the limitations and the need for more studies to understand better the role of body checking behaviour in Brazil, this study provided satisfactory initial evidence of the psychometric validity of the BCQ in Brazil. This is important due to the limited quantity of psychometric validated scales for body image investigation [50] for Brazilian research. However, this is also important for cross-cultural studies, since this can now be used as an adequate measure for data collection in countries where body culture is evident and salient. Finally, the presented results suggest that physical educators and nutritionists in Brazil should be assessing body checking and body image behaviour more closely in their patient/client physical exercise and dieting programs.
